# Impaired redox adaptation through SLC38A5-dependent glutamine metabolism contributes to melatonin-mediated anoikis sensitivity and metastasis suppression in bone cancer

**DOI:** 10.7150/ijbs.133256

**Published:** 2026-05-11

**Authors:** Bao Tran Nguyen, Wei-Cheng Chen, Yi-Chin Fong, Chih-Yuan Ko, Hsien-Te Chen, Chin-Jung Hsu, Kieu Thanh Huynh, Wei-Chien Huang, Shun-Fa Yang, Chih-Hsin Tang

**Affiliations:** 1 Graduate Institute of Biomedical Sciences, China Medical University, Taichung, Taiwan.; 2 Department of Medicine, MacKay Medical University, New Taipei, Taiwan.; 3 Division of Sports Medicine & Surgery, Department of Orthopedic Surgery, MacKay Memorial Hospital, Taipei, Taiwan.; 4 Department of Sports Medicine, College of Health Care, China Medical University, Taichung, Taiwan.; 5 Department of Orthopedic Surgery, China Medical University Hospital, Taichung, Taiwan.; 6 Department of Orthopedic Surgery, China Medical University Beigang Hospital, Yunlin, Taiwan.; 7 School of Chinese Medicine, China Medical University, Taichung, Taiwan.; 8 Center of Molecular Medicine, China Medical University Hospital, Taichung, Taiwan.; 9 Department of Medical Laboratory Science and Biotechnology, Asia University, Taichung, Taiwan.; 10 Institute of Medicine, Chung Shan Medical University, Taichung, Taiwan.; 11 Department of Medical Research, Chung Shan Medical University Hospital, Taichung, Taiwan.; 12 Department of Pharmacology, School of Medicine, China Medical University, Taichung, Taiwan.; 13 Chinese Medicine Research Center, China Medical University, Taichung, Taiwan.

**Keywords:** bone cancer, glutamine metabolism, metastasis, SLC38A5, anoikis resistance, ROS

## Abstract

Malignant bone cancer, primarily osteosarcoma and chondrosarcoma, are highly aggressive neoplasms originating from bone tissue and characterized by a strong propensity for lung metastasis. Tumor cells evade anoikis through reactive oxygen species (ROS)-mediated redox homeostasis, which modulates signaling cascades that promote proliferation, survival, and metastatic invasion into distant sites. Melatonin, synthesized primarily by the pineal gland, has been implicated in cancer prevention and therapy due to its inhibitory effects on bone cancer growth and progression. Little is known about the mechanisms underlying anoikis resistance in bone cancer cells or whether melatonin can therapeutically modulate this process. We demonstrated that glutamine metabolism is essential for bone cancer cells to maintain anoikis resistance. Melatonin treatment disrupted glutamine metabolism and altered redox homeostasis, as evidenced by increased ROS accumulation and reduced NADPH/NADP⁺ ratios under anchorage-independent conditions. Notably, solute carrier family 38 member 5 (SLC38A5), a glutamine transporter, was identified as a critical regulator of bone cancer progression, with higher SLC38A5 expression correlating with poorer clinical outcomes. Melatonin suppressed SLC38A5 expression and attenuated anoikis resistance through inhibition of the PI3K-Akt signaling pathway. Consistently, reduced SLC38A5 expression was associated with decreased lung metastasis in melatonin-treated groups in an orthotopic mouse model. Collectively, our findings reveal a previously unrecognized role of melatonin in modulating glutamine-dependent redox balance and anoikis resistance in bone cancer. This study highlights SLC38A5-mediated glutamine metabolism as a critical determinant of metastatic potential and supports melatonin and SLC38A5 as promising therapeutic targets for osteosarcoma and chondrosarcoma.

## Introduction

Bone cancer is a relatively rare and complex cancer, comprising under 1% of all cancer diagnoses in the United States each year 1. Bone cancer is mainly categorized by the type of cell it originates from: osteosarcoma (the most common sarcoma), chondrosarcoma, and Ewing sarcoma 1, 2. The standard treatment for bone cancer predominantly involves a neoadjuvant protocol utilizing cisplatin, doxorubicin, methotrexate, and/or additional chemotherapeutic agents, succeeded by limb-sparing surgery or amputation; nevertheless, around 30% of patients demonstrate resistance to treatment 3. Chondrosarcoma, the second most prevalent primary bone malignancy, it exhibits greater resistance to chemotherapy than other bone cancers due to its distinct transcriptional landscape. This resistance includes poor responsiveness to neoadjuvant chemotherapy regimens commonly used in osteosarcoma treatment 4. Osteosarcoma and chondrosarcoma lesions often share similarities in anatomical location and their potential for pulmonary metastasis 5, 6; therefore, understanding the distinct molecular mechanisms underlying therapeutic resistance in each is essential for developing more effective, targeted treatment strategies. Although emerging research implicates the advantage of bone cancer treatment based on accurate staging, clinical outcomes have not improved much in over three decades 1. Consequently, there is a pressing need to identify and develop new therapeutic approaches for these aggressive cancers.

Resistance to anoikis, which refers to apoptosis triggered by detachment from the extracellular matrix, is a pivotal event in metastatic progression. This adaptation enables circulating tumor cells (CTCs) to remain viable and establish colonies within distant premetastatic niches 7. Anchorage-independent conditions enhance cancer cell migration and invasion and may facilitate CTC extravasation 8. To survive extracellular matrix detachment, cancer cells rely on metabolic and redox adaptation mechanisms that support redox buffering capacity and limit oxidative stress, thereby promoting anoikis resistance and metastatic progression 7. Elucidating the mechanisms that govern anoikis resistance will deepen our understanding of cancer metastasis and may reveal potential therapeutic targets to prevent tumor cell dissemination and metastatic progression 7. In bone cancer, the engagement of downstream signaling cascades has frequently been implicated in sustaining anoikis resistance. Although the mechanisms have been investigated across different bone cancer subtypes, the shared molecular features underlying anoikis resistance in these malignancies remain insufficiently defined. In cancer, metabolic reprogramming is a key feature that enables tumor progression, driving a dependence on specific nutrients to support continued proliferation and metastasis 9. Cancer cells upregulate processes such as aerobic glycolysis and glutaminolysis to meet these demands to sustain their rapid growth 10. Anoikis resistance is associated with various metabolic processes, including amino acid metabolism, EGFR signaling activation, fatty acid oxidation, pentose phosphate pathway flux, glutamine catabolism, lactate dehydrogenase A activity, and oleic acid metabolism, all of which enhance tumor cells' metastasis 7. However, the contribution of metabolism in chondrosarcoma and osteosarcoma cell anoikis resistance remains unclear. The metabolic utilization of glutamine, together with associated pathways-encompassing transporter-mediated uptake, glutaminase-driven catabolism, aminotransferase activity, and regulation of cellular redox status-constitutes a fundamental requirement for the persistence and growth of malignant cells 11. A previous study reported that under non-adherent conditions, malignant cells have been observed to sustain viability by redirecting their metabolic reliance from glucose toward glutamine-dependent energy generation 12. In addition, knockdown of glutamate dehydrogenase 1 (GDH1) not only limits tumor proliferation but also enhances anoikis sensitivity and restrains metastatic progression 10. Despite these findings, the contribution and specific functions of glutamine metabolism in regulating anoikis resistance in bone cancer remain poorly defined.

Melatonin is increasingly recognized as an anti-tumor molecule across a wide variety of malignancies 13. There is substantial evidence regarding its effects on osteosarcoma 14 and chondrosarcoma 15 via multiple pathways. However, the precise mechanisms underlying its influence on metabolic regulation and anoikis resistance in bone cancer are not yet fully understood. In this study, we performed RNA sequencing (RNA-seq) analysis to investigate metabolic alterations in bone cancer cells treated with melatonin under anchorage-independent conditions. We identified the glutamine transporter SLC38A5 as one of the most significantly downregulated genes following melatonin treatment. Consistent with disruption of glutamine-dependent metabolic adaptation, melatonin treatment was associated with increased ROS accumulation and altered NADPH/NADP⁺ redox status under anchorage-independent conditions. Notably, elevated SLC38A5 expression was associated with poorer clinical outcomes in patients with bone cancer. Functional analyses revealed that pharmacological inhibition of SLC38A5 markedly impaired anoikis resistance, at least in part through suppression of PI3K-Akt signaling. Furthermore, melatonin treatment significantly reduced lung metastasis of bone cancer cells *in vivo*, an effect that was accompanied by decreased SLC38A5 expression. Collectively, our findings uncover a previously unrecognized role of melatonin in disrupting glutamine-dependent redox adaptation required for anchorage-independent survival, thereby sensitizing bone cancer cells to anoikis and limiting metastatic progression. By linking metabolic transport, redox buffering capacity, and metastatic competence, this study highlights melatonin and SLC38A5 as potential therapeutic targets in osteosarcoma and chondrosarcoma.

## Materials and Methods

### Anoikis resistance

Bone cancer cells (5 × 10^4^/well) were plated in 24-well plates coated with poly-HEMA to prevent adhesion and maintained for 24 h. Cells were then exposed to melatonin at concentrations of 0.1, 0.3, or 1 mM for a period of three days. Cell aggregates were visualized by z-stack imaging using an ImageXpress Pico Automated Imaging System (Molecular Devices, USA). The aggregates were then collected, enzymatically dissociated with trypsin, and viable cell numbers were determined using 0.4% trypan blue exclusion 15, 16. Data are shown as the percentage of viable cells compared to untreated controls.

### Soft agar colony formation assays

Three-dimensional growth was evaluated using a soft agar assay in which 1 × 10³ cells were suspended in 0.3% agarose (Invitrogen) supplemented with or without melatonin (0.1-1 mM). Wells were overlaid with 1 mL of complete growth medium, which was replenished every three days. After 21 days, colonies were stained using MTT and visualized by z-stack imaging with an ImageXpress Pico Automated Imaging System (Molecular Devices, USA). Colony growth was subsequently quantified using ImageJ.

### Bioinformatic analysis

Total RNA was extracted from JJ012 and 143B cells treated with or without melatonin (1 mM) under detachment conditions for RNA sequencing (RNA-Seq). The transcriptome sequencing process involved RNA extraction and quality control (QC), followed by library construction and purification, library QC and quantification, cluster generation, and finally high-throughput sequencing. Differentially expressed genes (DEGs) were defined using the thresholds of |log₂(fold change)| ≥ 0.58 and an adjusted *p*-value (false discovery rate, FDR) < 0.05. These DEGs were subsequently uploaded to the Database for Annotation, Visualization and Integrated Discovery (DAVID) and QIAGEN Ingenuity Pathway Analysis (IPA) for functional enrichment analysis 17, 18.

The published GSE184118 dataset comprises single-cell RNA sequencing (scRNA-seq) data from chondrosarcomas of varying histological grades, as well as from enchondroma (a benign cartilage tumor) and chondroblastic osteosarcoma. Gene expression levels of SLC38A5 and GLS were analyzed and visualized using Loupe Browser software.

### Molecular docking

The three-dimensional structure of human SLC38A5 (AF-Q8WUX1-F1) was obtained from the AlphaFold Protein Structure Database. The structure of melatonin was retrieved from the Protein Data Bank (PDB ID: 5I8F). Molecular docking was performed using the CDOCKER module implemented in BIOVIA Discovery Studio 2022 (DS2022). The top-ranked binding pose was selected based on the CDOCKER interaction energy score. Protein-ligand interactions were further analyzed and visualized using the 2D interaction diagram and 3D visualization tools in DS2022.

### Glutamine assay

Cells were harvested, lysed, and prepared for glutamine quantification using the Glutamine Detection Assay Kit (ab197011; Abcam, Cambridge, UK) following the manufacturer's protocol. Briefly, clarified lysates were incubated with the supplied reagents, and glutamine levels were measured colorimetrically using a standard curve generated in parallel. To account for variations in cell number and protein yield, glutamine content was normalized to each sample's total protein concentration, determined by the bicinchoninic acid assay. Data were expressed as relative glutamine uptake compared with control groups.

### Seahorse XFe24 Mito stress assay

Bone cancer cells (1 × 10^4^) were seeded onto Seahorse XF microplates and incubated overnight after 3 days of suspension culture. Cells were pre-equilibrated for 1 h in Seahorse XF Assay Medium supplemented with 10 mM glucose, 1 mM sodium pyruvate, and 2 mM glutamine under CO₂-free conditions at 37 °C. Mitochondrial respiration was subsequently measured using a Seahorse XF Analyzer (Agilent) following sequential injections of oligomycin (1 µM), FCCP (1 µM), and rotenone/antimycin A (0.5 µM). Oxygen consumption rate (OCR) values were obtained and analyzed with Wave software (Agilent) 19.

### Intracellular ROS measurement

Bone cancer cells (5 × 10^4^) were cultured under anchorage-independent conditions and treated with melatonin (1 mM) for 3 days. To assess the functional role of SLC38A5, parallel experiments were performed in cells with or without SLC38A5 overexpression. Intracellular reactive oxygen species were detected using either 5,6-carboxy-2',7'-dichlorofluorescein diacetate (DCFDA / H2DCFDA - Cellular ROS Assay Kit; Abcam, ab113851). Cells were incubated with 10 μM DCFDA at 37 °C in the dark for 1 h. Following incubation, cells were lysed in RIPA buffer, and fluorescence intensity was quantified using a microplate reader with excitation/emission wavelengths set at 485/535 nm. Fluorescence values were normalized to total protein content to account for variations in cell number.

### NADPH/NADP+ assay

Melatonin-treated suspension bone cancer cells, with or without SLC38A5 overexpression, were collected to measure total NADP and NADPH levels using the NADP/NADPH Assay Kit-WST (Dojindo, N510) according to the manufacturer's manual.

### Transfection

In order to create cell lines that overexpress SLC38A5, JJ012 and 143B cells were utilized, Lipofectamine 2000 from Thermo Fisher Scientific Inc., Illinois, USA, transfected with 1 μg/μL of either a control pcDNA3.1(+) vector or a pcDNA3.1(+) construct encoding SLC38A5 (MD Bio Inc., Montgomery County, MD, USA). Geneticin at 200 μg/mL (G418; Life Technologies) was applied to select cells 24 h post-transfection under standard culture conditions. Stable clones were subsequently isolated following the procedure outlined in our prior study 15.

### Animal model

To further investigate the *in vivo* role of melatonin in bone cancer lung metastasis, 5 × 10^6^ cells of JJ012 and 2 × 10^5^ cells of 143B stably expressing luciferase (Luc) were injected into the tibia of 6-week-old CAnN.Cg-Foxn1nu/Crl (BALB/c Nude) male mice (approximately 22 grams each). One week later, all the mice were randomly assigned to three groups (6 mice per group; sample size was determined based on similar previous studies 15, 20). Melatonin was administered by intraperitoneal (IP) injection three times a week at low (20 mg/kg) or high (60 mg/kg) doses, or phosphate-buffered saline (PBS) as vehicle, at the same frequency. Approximately eight weeks post-treatment, tumor growth in the tibia was monitored using a Xenogen IVIS Imaging System 200 (PerkinElmer, Waltham, MA, USA). Mice were subsequently euthanized by CO₂ inhalation, and lung and blood samples were collected for analysis of metastatic burden. The mice were maintained under anesthesia with 1.5% isoflurane during the experiment and housed under specific pathogen-free conditions with free access to food and water under a 12 h light/dark cycle. Humane endpoints were predefined, and animals were euthanized if tumor burden exceeded 10% of total body weight, if tumor growth impaired mobility, or if body weight decreased by more than 25% relative to baseline.

### Immunohistochemistry (IHC) staining

The OS805a tissue array (US Biomax; Rockville, MD, USA) was processed following an optimized protocol adapted from our protocol in the previous publication 15. In brief, paraffin was removed from the sections using xylene, after which the samples were rehydrated through a descending ethanol gradient. Antigen retrieval was performed using a citrate-based retrieval buffer, and endogenous peroxidase activity was neutralized. Sections were then incubated overnight at 4 °C with the primary anti-SLC38A5 antibody (1:50 dilution). The following day, a secondary antibody was applied for 30 min. Signal detection was carried out using 3,3'-diaminobenzidine (DAB), followed by counterstaining with hematoxylin, graded dehydration, and coverslip mounting. SLC38A5 expression levels were scored according to the previously reported evaluation criteria 15, 20.

### Statistical analysis

All quantitative analyses were performed using GraphPad Prism software (version 10.3.1). For pairwise comparisons, statistical significance was evaluated using the Student's *t*-test. Analyses involving more than two groups were conducted with one-way ANOVA, and Tukey's multiple comparison procedure was applied as a post hoc test. Data are expressed as mean ± standard deviation (SD) derived from a minimum of three independent experiments. *p* < 0.05 indicates a statistically significant difference.

## Results

### Melatonin inhibits bone cancer cell anoikis resistance and metastasis progression

**Figure 1:**
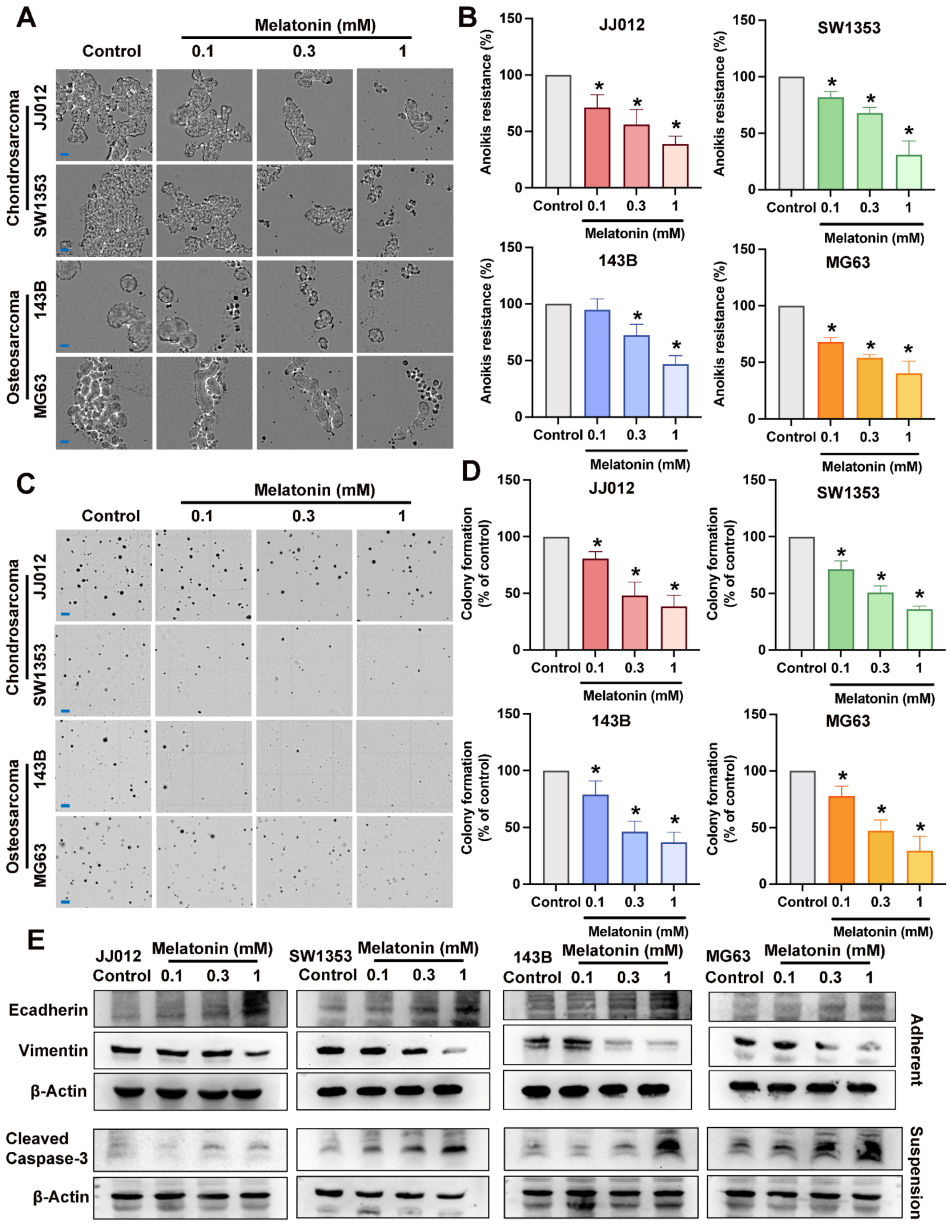
** Pharmacology of melatonin in bone cancer cell anoikis resistance and metastasis *in vitro*. (**A&B) Chondrosarcoma cells (JJ012, SW1353) and osteosarcoma cells (143B, MG63) were subjected to various concentrations of melatonin (0.1, 0.3, 1 mM) for an interval of 3 days under suspension conditions. Alive cells were calculated and expressed as a percentage of untreated control cells (scale bar = 150 µm). (C&D) Bone cancer cells were cultured in a medium with 0.3% agarose, with or without specified amounts of melatonin; clonogenicity was assessed after 21 days (scale bar = 500 µm) (E) Bone cancer cells incubated with different concentrations of melatonin (0.1, 0.3, 1 mM) for 24 h; markers associated with EMT-like plasticity (E-cadherin and Vimentin) and apoptosis marker (cleaved Caspase-3) were examined by Western blot. *p < 0.05.

Several different signaling pathways were shown to differ significantly between floating and adherent cells 21. Previous studies have indicated that melatonin exerts anticancer effects via several pathways 13, 14; however, its inhibitory role on aggregate bone cancer cells remains unclear. To examine the anoikis resistance and anchorage-independent inhibition of melatonin in bone cancer, chondrosarcoma cells (JJ012, SW1353) and osteosarcoma cells (143B, MG63) were treated with melatonin in different doses (0.1, 0.3, 1 mM) 15. Melatonin markedly suppressed cell growth and anoikis resistance across all cell lines in a dose-dependent manner (Fig. 1A-D). Anoikis resistance characterizes oncogenic epithelial-mesenchymal transition (EMT) and is essential for metastasis 7. The reduction of Vimentin and augmentation of E-cadherin expression by different doses of melatonin in both chondrosarcoma and osteosarcoma cell lines (Fig. 1E) suggested that melatonin may suppress EMT-like phenotypic plasticity in bone cancer cells. The activation of caspase-3 induces distinct alterations in the morphology and biochemistry of apoptotic cells, recognized as the hallmarks of apoptosis 22. The cleaved Caspase-3 expression in the melatonin-treated groups was significantly increased, indicating that melatonin reverses the anoikis resistance ability by inducing apoptosis of bone cancer cells, as shown in Figure 1E. Therefore, melatonin may serve as a potential therapeutic agent against bone cancer cell anoikis resistance and metastasis.

### Glutamine metabolism contributes to bone cancer anoikis resistance

**Figure 2:**
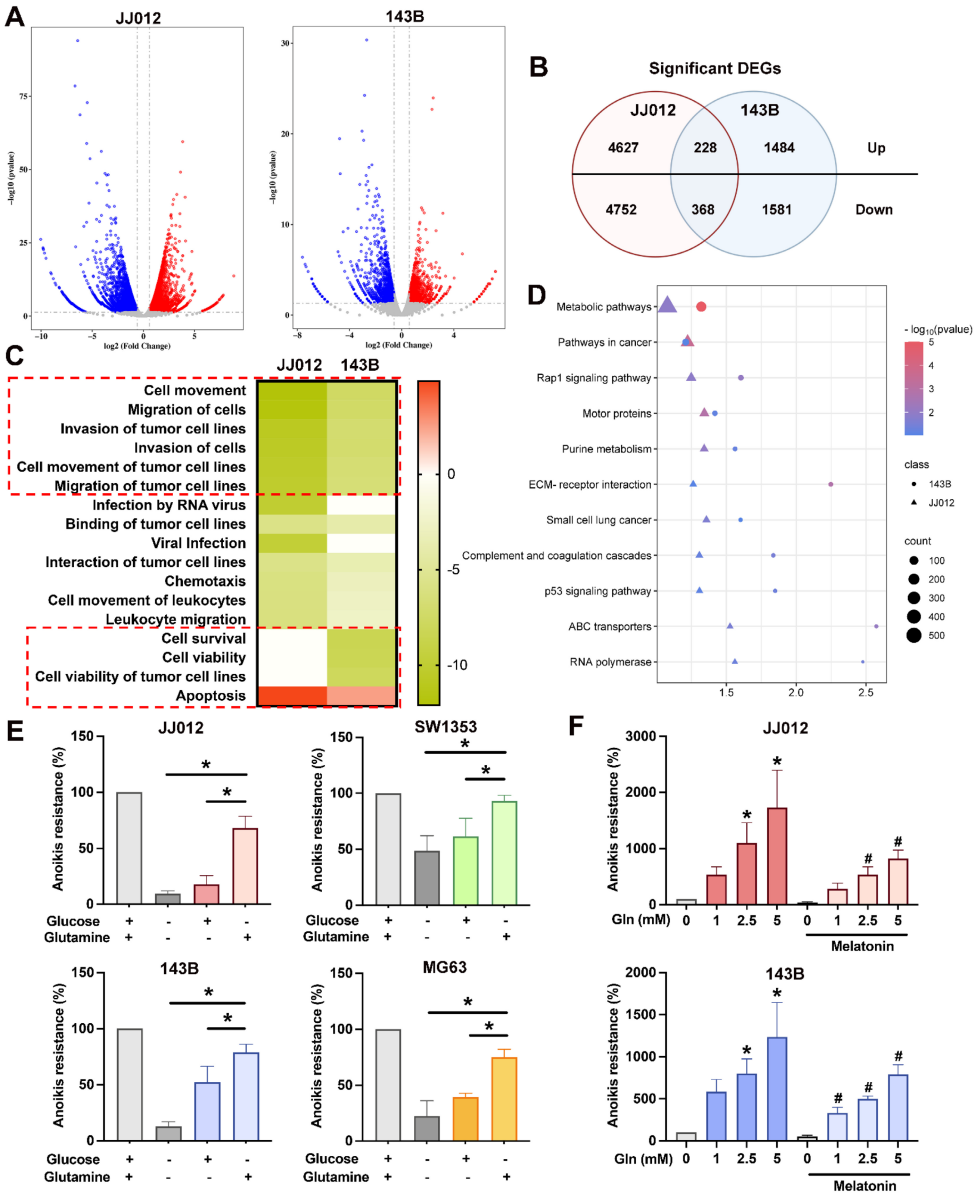
** Melatonin regulates metabolic pathways in bone cancer cell anoikis resistance.** JJ012 and 143B suspense cells were treated with melatonin (1 mM) for 3 days. (A&B) The significant difference genes were presented by the volcano plot, and similar genes were identified. (C) Histogram of diseases enriched from target genes of significantly down-regulated genes following melatonin treatment, analyzed using IPA. (D) DAVID-based enrichment analysis of DEGs identifies pathways significantly altered by melatonin. JJ012 and 143B cells were maintained in a culture medium with (E) different components or (F) different glutamine concentrations with or without melatonin (1 mM) for 3 days, and anoikis resistance abilities were analyzed. *p < 0.05.

To elucidate the potential mechanisms underlying the functional effects of melatonin in suspension state, JJ012 and 143B cells treated with 1 mM melatonin from suspension culture were subjected to RNA sequencing. The analysis indicates multiple genes with significant differences changed by melatonin (Fig. 2A). A total of 596 overlapping genes (228 upregulated and 368 downregulated) were identified between the two cell lines (Fig. 2B). Functional pathway analysis using Ingenuity Pathway Analysis (IPA) revealed that melatonin treatment was associated with significant inhibition of pathways related to cell movement, migration, and invasion, as well as cell survival and viability (Fig. 2C). In contrast, apoptosis-related pathways were predicted to be activated. Moreover, KEGG pathway enrichment analysis using DAVID demonstrated that the DEGs were predominantly enriched in metabolic and cancer-related pathways (Fig. 2D). Glucose and glutamine are among the most rapidly consumed nutrients in cancer cells and serve as the two primary carbon sources, both feeding into the tricarboxylic acid (TCA) cycle to support energy production 11, 23. Therefore, we investigated which nutrient resources contribute to the anoikis-resistant abilities of bone cancer cells. In comparison to glucose, glutamine is more essential for the survival of chondrosarcoma and osteosarcoma cells in suspension, as shown in Figure 2E. Interestingly, cultured cells in detachment conditions with different glutamine concentrations with or without melatonin indicated that glutamine significantly promotes bone cancer cell anoikis resistance and reverses the inhibition effect of melatonin (Fig. 2F). These findings highlight the potential role of glutamine in modulating anoikis resistance in cancer, warranting further investigation into its mechanisms and potential therapeutic implications.

### Melatonin suppresses SLC38A5 and glutamine metabolism in chondrosarcoma and osteosarcoma

**Figure 3:**
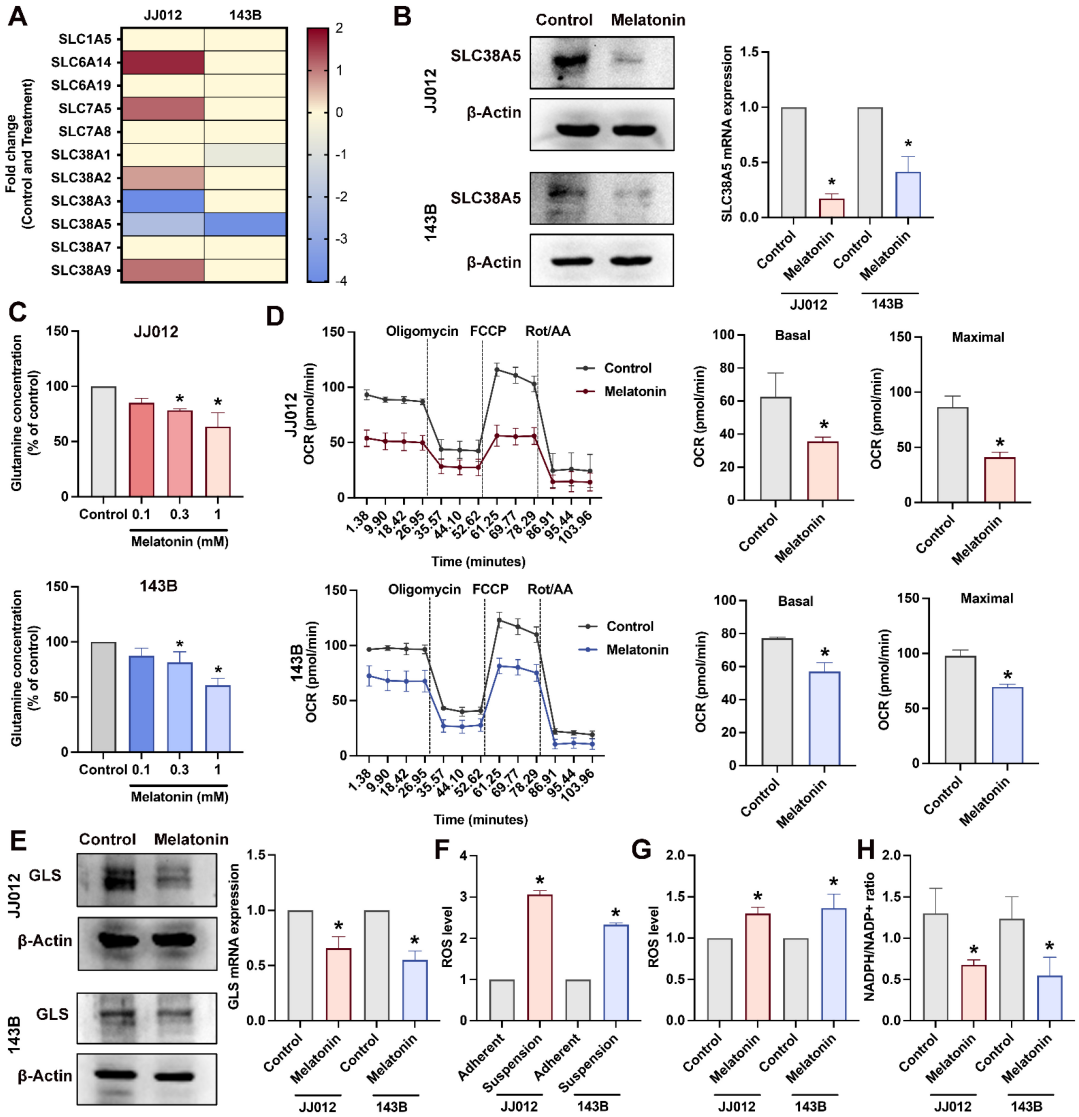
** The effects of melatonin on glutamine transport, mitochondrial respiration, and redox homeostasis in bone cancer cells.** (A) RNA-seq analysis showing fold changes in glutamine transporter expression following melatonin treatment. (B) Cells were treated with melatonin (1 mM) for 3 days, and SLC38A5 protein and mRNA expression levels were analyzed by Western blotting and qPCR, respectively. (C) Intracellular glutamine concentrations were measured after treatment with increasing concentrations of melatonin (0.1, 0.3, and 1 mM). (D) Oxygen consumption rate (OCR) was assessed using a Seahorse XF analyzer. Cells cultured in suspension were seeded onto Seahorse plates and allowed to adhere for 24 h prior to analysis; melatonin was added 2 h before the assay. Basal and maximal respiration were determined following sequential injections of oligomycin, FCCP, and rotenone/antimycin A. (E) GLS protein expression and mRNA levels were evaluated after 3 days of melatonin treatment under suspension conditions. (F-G) Intracellular ROS levels were measured in adherent and suspension-cultured cells following melatonin treatment. (H) The NADPH/NADP⁺ ratio was determined to assess cellular redox status.*p < 0.05.

Upregulation of glutamine transporters, including members of the SLC1, SLC7, and SLC38 families, is essential for cancer cells to sustain an increased demand for glutamine uptake from the extracellular milieu 11, 24. Our RNA-seq analysis revealed that melatonin significantly altered the expression of multiple glutamine transporters in bone cancer cells. Among these, SLC38A5 was prioritized for further investigation due to its consistent and marked downregulation across both JJ012 and 143B cell lines relative to other glutamine transporters (Fig. 3A). Melatonin-mediated suppression of SLC38A5 at both the mRNA and protein levels was validated in JJ012 and 143B cells (Fig. 3B). Given that SLC38A5 functions as a glutamine transporter, we employed a glutamine assay to assess whether SLC38A5 regulates glutamine utilization in chondrosarcoma and osteosarcoma cells. Consistent with this, glutamine levels were dose-dependently reduced in bone cancer cells following melatonin treatment (Fig. 3C). After entering the cell, glutamine undergoes a series of metabolic reactions within the mitochondria, which are integral to mitochondrial function and essential for various cellular processes 25. To investigate the effects of melatonin on mitochondrial activity, we treated cells with melatonin and assessed mitochondrial function using the Seahorse XFe24 Analyzer by measuring the oxygen consumption rate (OCR), providing insights into cellular bioenergetics. Acute melatonin treatment resulted in a rapid decline in key mitochondrial function parameters, including the basal OCR and maximal respiratory capacity (Fig. 3D). To further assess whether melatonin inhibits glutamine metabolism in bone cancer cells, we examined glutaminase (GLS) expression, a key enzyme in glutamine metabolism that is highly expressed in bone cancer, including chondrosarcoma and osteosarcoma patient samples (Supplementary Fig. S1). As expected, melatonin reduced GLS protein expression and mRNA level (Fig. 3E) in both JJ012 and 143B cells.

Reactive oxygen metabolites are crucial for sustaining cancer cell metabolism, genetic instability, proliferation, and angiogenesis. Moreover, reactive oxygen species (ROS) function as signaling molecules that might enhance cell survival at the expense of apoptosis 26. Consistent with this notion, JJ012 and 143B cells exhibited elevated intracellular ROS levels under anchorage-independent conditions compared with their adherent control cultures (Fig. 3F). Contrary to its classical antioxidant role, prolonged melatonin treatment further increased ROS accumulation in bone cancer cells cultured in suspension relative to untreated controls (Fig. 3G). To determine whether these changes in ROS were accompanied by alterations in redox buffering capacity, we next examined the NADPH/NADP⁺ ratio. Notably, melatonin treatment under suspension conditions was associated with a significant reduction in the NADPH/NADP⁺ ratio in both JJ012 and 143B cells (Fig. 3H), suggesting impaired cellular redox buffering. Together, these results indicate that melatonin-induced ROS accumulation in anchorage-independent bone cancer cells is accompanied by diminished NADPH availability. All these findings prompted us to further investigate the involvement of SLC38A5, a glutamine transporter, in melatonin-associated redox alterations and anoikis resistance in bone cancer cells.

**Figure 4:**
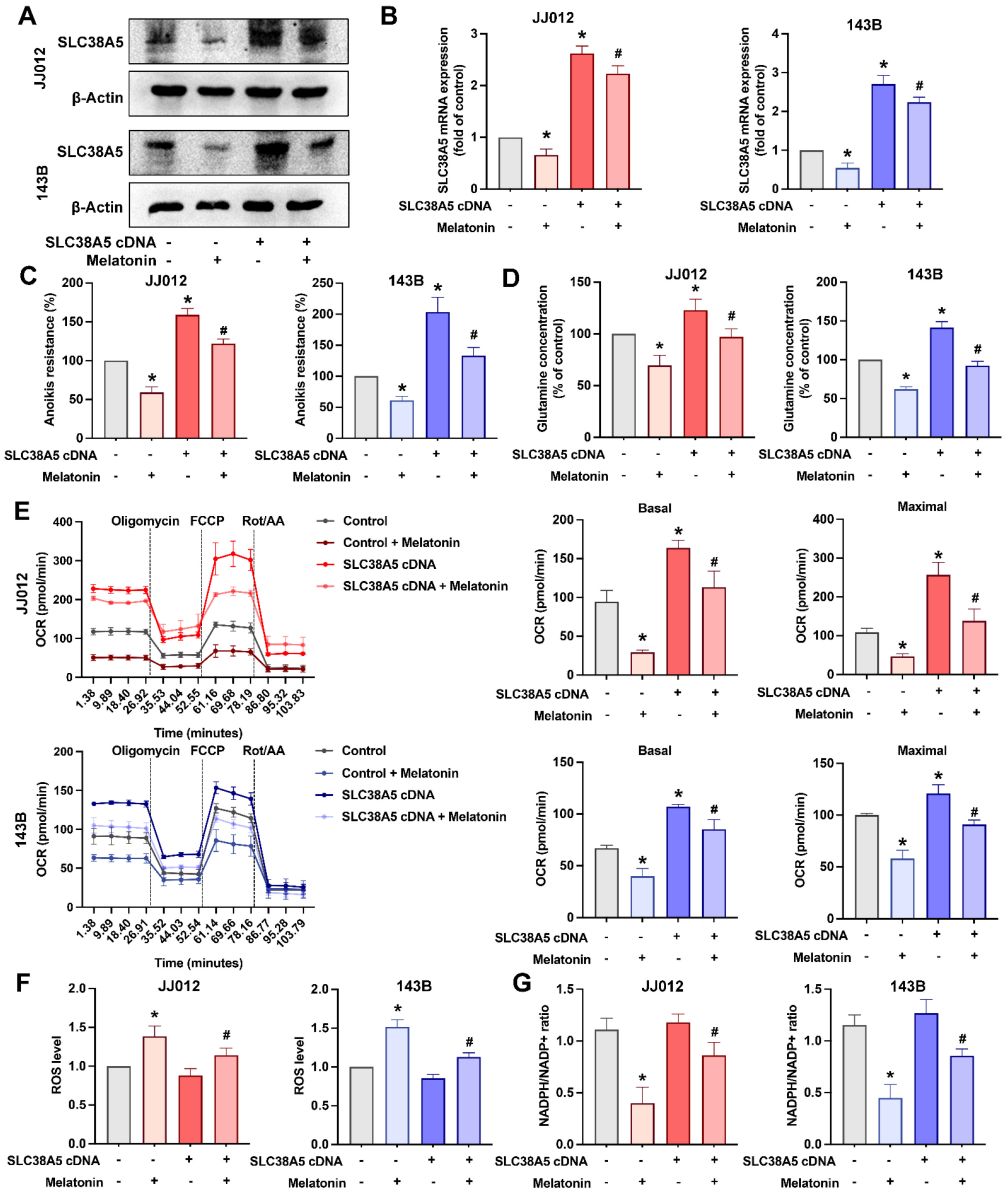
** Melatonin counteracts SLC38A5 overexpression-mediated anoikis resistance and metabolic reprogramming in bone cancer cells.** JJ012 and 143B cells were transfected with an empty vector or an SLC38A5-overexpressing construct (pcDNA3.1/SLC38A5), followed by melatonin treatment. (A) SLC38A5 protein expression was analyzed by immunoblotting to confirm overexpression and the inhibitory effect of melatonin. (B-C) Parental and SLC38A5-overexpressing cells were treated with melatonin (1 mM) for 3 days under suspension conditions, and anoikis resistance and intracellular glutamine levels were assessed. (D) Intracellular glutamine concentrations were measured to evaluate SLC38A5-mediated glutamine uptake. (E) Mitochondrial respiration was analyzed by Seahorse XF assay in parental and SLC38A5-overexpressing cells following melatonin treatment. Basal and maximal oxygen consumption rates (OCR) were calculated after sequential injection of oligomycin, FCCP, and rotenone/antimycin A. (F) Intracellular ROS levels were determined. (G) The NADPH/NADP⁺ ratio was measured to assess cellular redox balance. Data are presented as mean ± SD. *p < 0.05 compared with the control group; #*p* < 0.05 compared with SLC38A5 overexpression group.

We next examined the role of SLC38A5 in melatonin-mediated regulation of anoikis resistance in bone cancer cells. Overexpression of SLC38A5 markedly attenuated the inhibitory effects of melatonin on anoikis resistance, intracellular glutamine levels, and glutamine metabolism in both chondrosarcoma and osteosarcoma cell lines (Fig. 4A-E). Notably, SLC38A5 overexpression did not appreciably reduce basal ROS levels under anchorage-independent conditions, where oxidative stress is intrinsically elevated. However, restoration of SLC38A5 expression partially reversed melatonin-associated ROS accumulation and NADPH/NADP⁺ depletion (Fig. 4F&G). These findings indicate that melatonin suppresses anoikis resistance, metabolic adaptation, and redox homeostasis in bone cancer cells, at least in part, by downregulating SLC38A5.

### SLC38A5 has high expression in bone tumors

**Figure 5:**
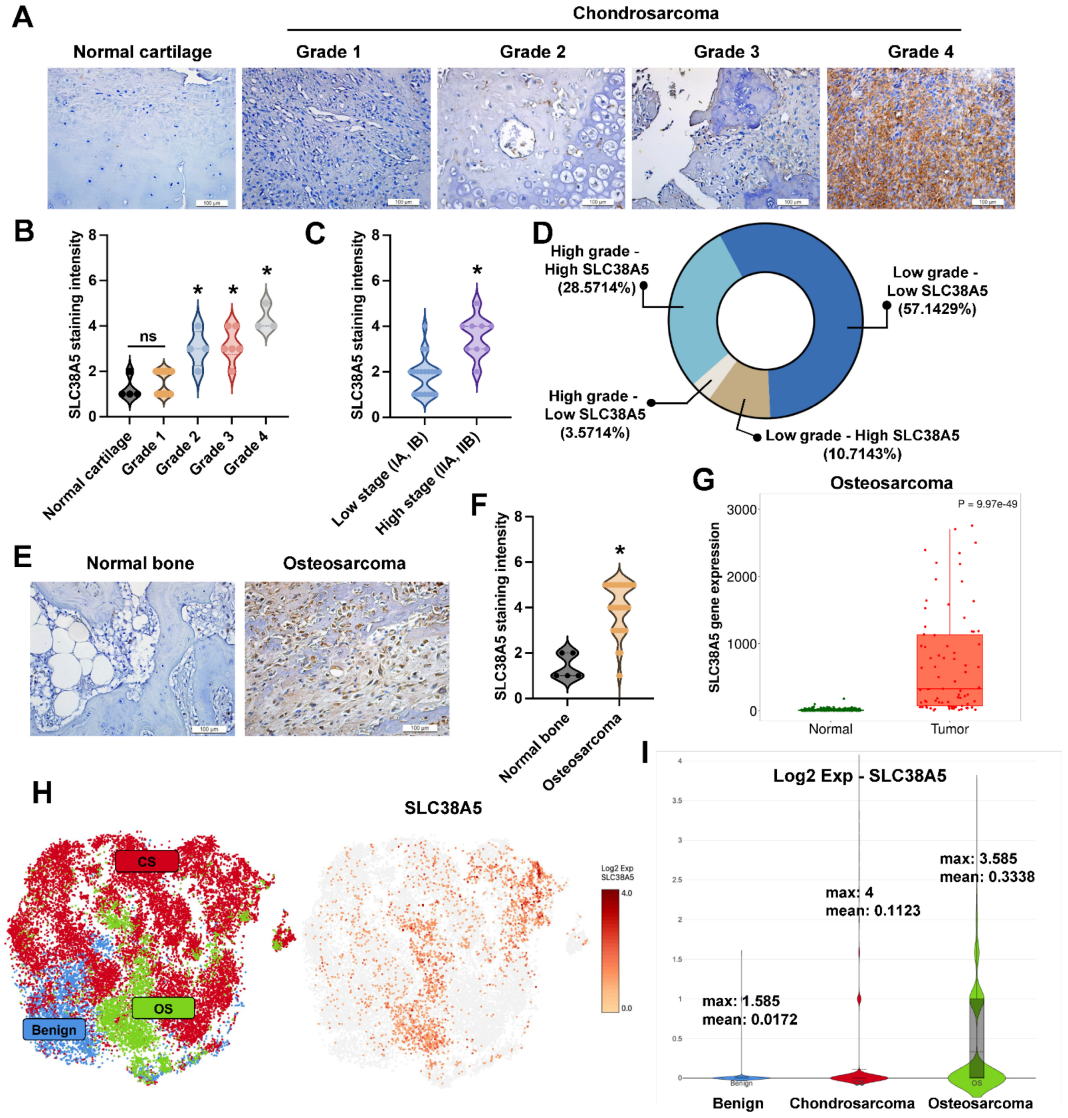
** SLC38A5 is upregulated in chondrosarcoma and osteosarcoma and is implicated in cancer progression.** (A) Representative immunohistochemical staining images of SLC38A5 in normal cartilage and chondrosarcoma tissues of different grades (Grade 1-4) (scale bar = 50 μm). (B-C) Quantitative data are depicted by violin plots of normal cartilage, tumor grade, and stage. (D) The pie graphs indicate the percentages of samples under different conditions. (E-G) Representative IHC images and quantification of SLC38A5 staining intensity in normal bone and osteosarcoma tissues, along with gene expression analysis from the TNMplot database. (H&I) Single-cell RNA seq depicts SLC38A5 expression in bone tumors compared to the benign. *p < 0.05.

Some research indicated that SLC38A5 has high expression in breast cancer 27 and pancreatic adenocarcinoma 28. To investigate the SLC38A5 expression in bone tumors, we detected SLC38A5 expression in patient samples using IHC staining. The expression of SLC38A5 in chondrosarcoma tissue was compared to that in normal cartilage tissue, demonstrating that elevated levels of SLC38A5 expression correlate with higher grades and stages of the disease (Fig. 5A&B). Analysis of tissue samples by disease stage revealed that high-grade chondrosarcoma cases frequently exhibited elevated SLC38A5 expression, whereas low-grade cases predominantly showed lower expression (Fig. 5C). As shown in Fig. 5D, most high-grade tumors had high SLC38A5 levels, while a subset displayed low expression. Similarly, low-grade tumors were mostly associated with low SLC38A5 expression, with a minority showing high levels. These findings suggest a potential link between increased SLC38A5 expression and chondrosarcoma progression. Similarly, this protein shows significantly higher expression in osteosarcoma patients, as assessed by IHC staining and the TNMplot database (Fig. 5E-G). Furthermore, we used the GEO database to examine SLC38A5 expression. The GSE184118 provided the single-cell RNA-seq analysis of chondrosarcomas, osteosarcomas, and a benign. As expected, SLC38A5 is highly expressed in chondrosarcoma and osteosarcoma clusters (Fig. 5H&I). These results support the contention that SLC38A5 appears as a potential candidate to contribute to bone cancer progression.

### Melatonin induces bone cancer cells anoikis by targeting the PI3K and Akt pathway

**Figure 6:**
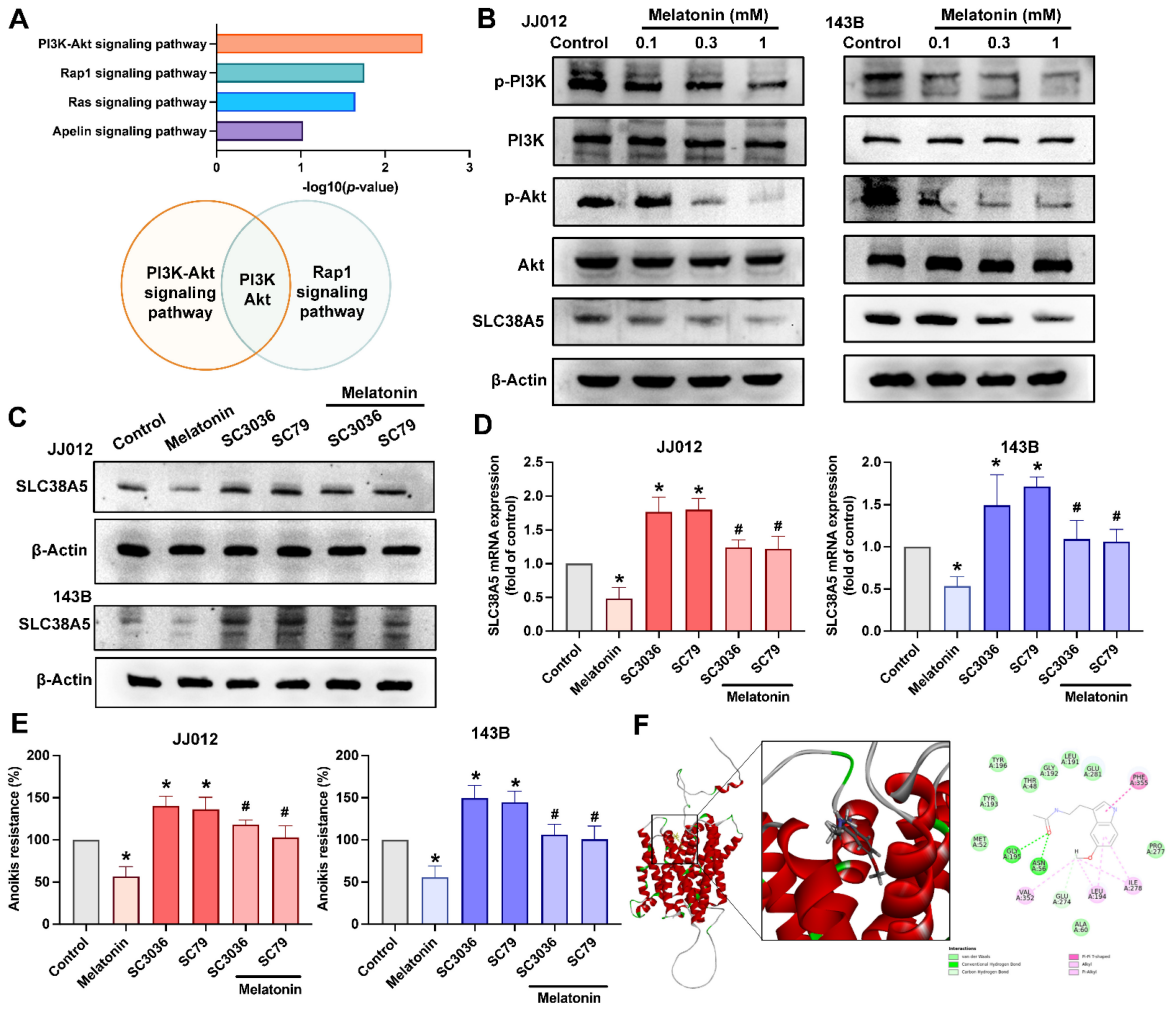
** Melatonin inhibits SLC38A5 expression by directly binding and via the PI3K-Akt signaling pathway.** (A) KEGG pathway enrichment analysis of RNA-seq-derived differentially expressed genes (DEGs) reveals significantly enriched pathways. (B) JJ012 cells were treated with different doses of melatonin (0.1, 0.3, and 1 mM) for 24 h, and the total and phosphorylated levels of PI3K, Akt, and the regulatory SLC38A5 were examined by immunoblotting. JJ012 and 143B were pretreated with PI3K and Akt activators for 30 min, followed by melatonin (1 mM) treatment for 24 h. (C&D) Western blotting and qPCR analysis indicating SLC38A5 protein and mRNA levels, and (E) anoikis resistance abilities were investigated. (F) The binding position of melatonin and SLC38A5 is analyzed by molecular docking. *p < 0.05.

KEGG pathway enrichment analysis revealed that the PI3K-Akt and Rap1 signaling pathways were among the most significantly enriched pathways following melatonin treatment, as determined by -log₁₀(*p*-value) ranking (Fig. 6A). Notably, shared components within the PI3K-Akt axis suggest a central role of this pathway in mediating melatonin-induced effects. The data in Figure 6B indicate that treating JJ012 and 143B cells with melatonin decreased PI3K and Akt phosphorylation and SLC38A5 expression dose-dependently. To investigate the regulatory effect of melatonin on SLC38A5 expression, we treated JJ012 and 143B cells with melatonin, SC3036 (PI3K activator), and SC79 (Akt activator), alone or in combination. Activation of the PI3K-Akt pathway induced SLC38A5 expression and anoikis resistance, whereas co-treatment with melatonin attenuated this induction (Fig. 6C-E). Since SLC38A5 is primarily involved in glutamine transport in tumor cells 25, we investigated whether melatonin directly interacts with SLC38A5. Molecular docking analysis predicted a binding interaction between melatonin and SLC38A5, with a docking energy value of -31.4458 kcal/mol, suggesting a potential direct association (Fig. 6F). Although these findings are based on *in silico* prediction, they raise the possibility that melatonin may, at least in part, modulate anoikis resistance and metastatic potential in bone cancer through regulation of SLC38A5 and the PI3K-Akt signaling pathway.

### Melatonin inhibits bone cancer lung metastasis *in vivo*

**Figure 7:**
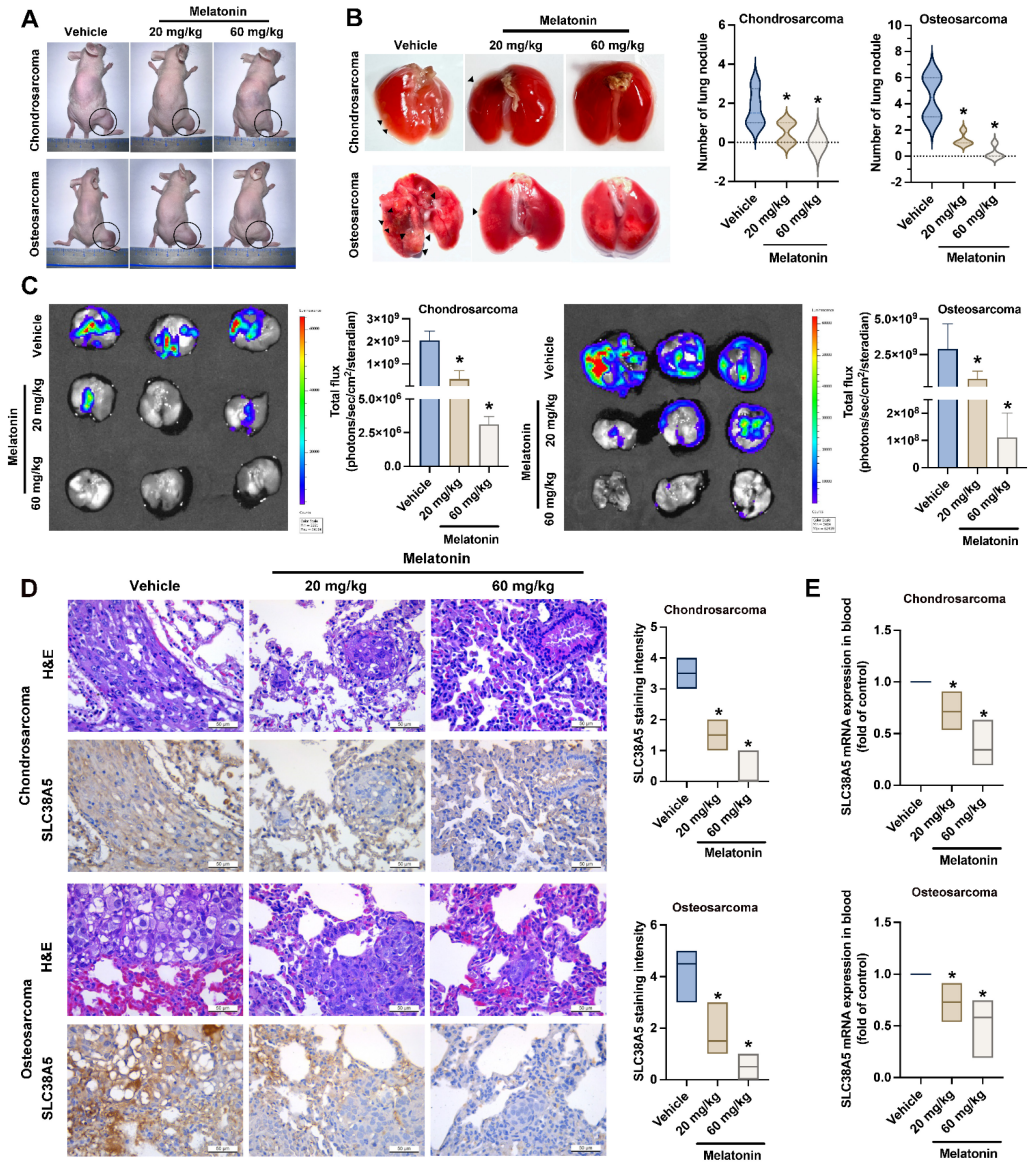
** Melatonin inhibits bone cancer cell anoikis resistance and metastasis to the lungs *in vivo*.** (A) Representative images of mice bearing chondrosarcoma or osteosarcoma tumors following treatment with control, low-dose melatonin, or high-dose melatonin. Tumor sites are circled. All animals (n = 6 per group) were euthanized at 8 weeks following melatonin administration (low dose: 20 mg/kg, high dose: 60 mg/kg). (B) Black arrowheads indicate metastatic lung nodules. Violin plots quantify the number of lung nodules per group. (C) At the specified intervals, the imaging signal intensity (photons/sec/cm^2^/steradian) was measured to determine luciferase activity using an IVIS® Spectrum *in vivo* imaging system. (D) Histology and levels of SLC38A5 expression in chondrosarcoma and osteosarcoma tumors under different settings were defined by using IHC staining and hematoxylin & eosin (H&E) staining. (E) Mouse blood samples were analyzed for levels of human SLC38A5 mRNA by qPCR. *p < 0.05.

To explore the* in vivo* inhibition effects of melatonin on lung metastasis, orthotopic bone cancer injection mice were administered melatonin every 3 days for a duration of 8 weeks. Our data indicated that while melatonin treatment did not significantly affect the growth of primary osteosarcoma tumors (Fig. 7A & Supplementary Fig. S2), it markedly suppressed lung metastasis in both chondrosarcoma and osteosarcoma groups, particularly at the high dose by the significant reduction of metastatic nodules (Fig. 7B). Consistently, bioluminescence imaging revealed a substantial decrease in metastatic tumor burden in the lungs of melatonin-treated mice, as indicated by reduced total photon flux in both cancer models (Fig. 7C). Immunohistochemical analysis further demonstrated a dose-dependent decrease in SLC38A5 protein expression in lung metastatic lesions in response to melatonin treatment (Fig. 7D). To confirm the melatonin-induced anoikis effect *in vivo*, we analyzed human SLC38A5 mRNA levels in mouse blood samples. The results demonstrated a consistent downregulation of SLC38A5 mRNA in the melatonin-treated group compared to the control group (Fig. 7E). These findings suggest that melatonin suppresses anoikis resistance in bone cancer and inhibits metastasis to the lungs by downregulating SLC38A5 expression in animal model.

## Discussion

Primary bone cancer, although rare, is associated with significant morbidity and mortality 1. Osteosarcoma and chondrosarcoma often arise within osseous tissue, and their lesions frequently exhibit similarities in location, appearance, and osteogenic texture. This complicates correct diagnosis and classification, and treatment options fluctuate significantly because of the varied efficacy of radiation and chemotherapy 5. There are essential indicators of a tenfold increase in the prevalence of both osteosarcoma and chondrosarcoma over the last two decades 29. Notably, approximately 15% of osteosarcoma patients and 8% of chondrosarcoma patients exhibited metastatic disease at the time of initial diagnosis 29, 30. Therefore, it is imperative to dedicate efforts toward the discovery of novel therapeutics and targets for early detection and management of primary bone cancer proliferation and metastasis. In our study, we nominate melatonin, an indolamine hormone that has been extensively investigated in the field of cancer research. Although melatonin's individual inhibitory effects are observed in both chondrosarcoma and osteosarcoma 14, 15, this is the first time identifying its common molecular targets is essential for a comprehensive understanding and potential therapeutic application in bone sarcomas and other types of sarcoma.

Anoikis is a vital inhibitory process that halts cancer metastasis. Investigating the molecular mechanisms that enable cells to evade anoikis will provide critical insights into the biology of metastasis and support the development of therapies aimed at preventing tumor cell survival and colonization at distant sites 7. In recent years, the investigation of dysregulated amino acid metabolism pathways in sarcomas has attracted heightened interest, revealing promising new insights into pathways that may represent tumor-specific vulnerabilities 31. Endo *et al.* showed that aggressive cancer cell lines preferentially utilize glutamine over glucose to sustain anchorage-independent survival 12. Despite these findings, the metabolic determinants underlying anoikis resistance in osteosarcoma and chondrosarcoma remain largely unexplored. To the best of our knowledge, our study was the first to demonstrate that bone cancer cells require glutamine rather than glucose to resist anoikis. Prior research has shown that glutamine deficiency suppresses osteosarcoma cell proliferation, and highly metastatic cells depend on glutamine as a crucial nutrient for cell growth both *in vivo* and *in vitro* 32. Moreover, glutamine metabolism has been shown to promote cisplatin resistance in chondrosarcoma cells by increasing NADPH production and limiting ROS accumulation 19. Our findings imply that glutamine contributes to osteosarcoma and chondrosarcoma cells to proliferate in suspension conditions and avoid detachment-induced cell death (Fig. 2). Therefore, the reported research and our study emphasize the important role of glutamine in bone cancer progression and metastasis.

Chondrosarcoma is generally highly resistant to radiation, whereas osteosarcoma exhibits radioresistance in certain instances 33. Existing literature suggests that glutamine, produced by skeletal muscle, may exhibit a dual role in sarcoma biology: promoting tumor growth through metabolic support and influencing the tumor's response to radiation therapy 34. Thus, pharmacological inhibition of glutamine metabolism could be viable for both chondrosarcoma and osteosarcoma treatment and may be combined with radiation therapy. Melatonin has emerged as a promising anticancer agent, demonstrating potential efficacy both as a standalone treatment and in combination with established chemotherapeutic regimens (ClinicalTrials.gov ID: NCT01858155, NCT00668707, NCT04137627). Our study indicated that melatonin inhibits bone cancer anoikis resistance and metastasis by reducing glutaminase and glutamine metabolism. Interestingly, glutaminase has elevated expression in the majority of chondrosarcoma cell lines relative to the control 35 and is associated with increased metastasis in osteosarcoma cells 32. To date, glutaminase inhibitor CB-839 is evaluated in osteosarcoma 32 and chondrosarcoma 35, and the clinical trial for a combination treatment approach in various cancer types (ClinicalTrials.gov ID: NCT03965845, NCT03163667, NCT03428217) 36, 37. Given the numerous clinical applications of melatonin as an adjuvant cancer therapy 38, we propose that a combination of melatonin, CB-839, and radiation therapy targeting glutamine metabolism may represent a promising therapeutic strategy for inhibiting bone cancer metastasis in the future.

Although melatonin is widely recognized for its antioxidant properties under physiological conditions, emerging evidence indicates that it exerts distinct redox effects at pharmacologically relevant concentrations in cancer cells 39, 40. At physiological to low pharmacological concentrations (10⁻⁸-10⁻⁶ M; nanomolar to low micromolar range), melatonin enhances intracellular antioxidant capacity by increasing glutathione levels and the activity of antioxidant enzymes, thereby reducing ROS accumulation 41. In contrast, at higher pharmacological concentrations (≥10⁻⁴-10⁻³ M; 0.1-1 mM), melatonin has been reported to induce ROS generation and promote cytotoxicity in multiple cancer models 39, 41. However, the redox consequences of melatonin treatment under anchorage-independent conditions remain incompletely understood. Peng *et al.* demonstrated that melatonin (100 µM) preserves cardiolipin integrity and protects against mitochondrial oxidative damage under conditions of mitochondrial stress, highlighting its classical antioxidant role in adherent cellular contexts in 143B cells 42. Interestingly, in our study, anchorage-independent culture was associated with elevated basal ROS levels compared with adherent conditions, and melatonin treatment at a higher dose and prolonged duration under suspension culture further augmented ROS accumulation, suggesting that cellular attachment status, together with treatment context, critically influences melatonin-induced redox responses. To our knowledge, concurrent assessment of ROS accumulation and NADPH/NADP⁺ redox status in anchorage-independent bone cancer cells following melatonin treatment has not been reported. Consistently, we observed a reduced NADPH/NADP⁺ ratio, indicating compromised redox buffering capacity, which is particularly relevant given the reliance of detached cancer cells on NADPH-producing pathways to withstand oxidative stress.

Cancer cells require an abundant supply of glutamine from the extracellular environment. To fulfill this requirement, they frequently upregulate glutamine transport systems, a process that can lead to local depletion of this nutrient 37. Within this context, the SLC38 family of transporters has been recognized as a major regulator of glutamine uptake in diverse malignancies. However, their functional significance in primary bone cancers has not been thoroughly characterized, leaving an important gap in understanding the metabolic adaptations that drive these tumors 37. SLC38A1 and SLC38A5 were indicated as potential targets in osteosarcoma cell migration 25, 43; interestingly, the role of SLC38 family transporters in chondrosarcoma was unclear. Since the latter is an invasive method and not always possible in bone sarcoma, a potential enhancement for this situation is the identification of molecular biomarkers during the early stages of the illness or even prior to its beginning 1. Our study discovered that SLC38A5 is a novel target of melatonin in osteosarcoma and chondrosarcoma (Fig. 3). Mechanistically, melatonin suppresses anoikis resistance in bone cancer cells, at least in part, through downregulation of SLC38A5 via the PI3K-Akt signaling pathway, with molecular docking analysis further suggesting a potential interaction between melatonin and SLC38A5 (Fig. 6). Moreover, the expression of SLC38A5 is positively correlated with bone cancer malignancy. Notably, restoration of SLC38A5 expression partially attenuated melatonin-induced ROS accumulation under suspension conditions, suggesting that glutamine transport contributes to redox homeostasis in anchorage-independent cells, although the precise mechanisms remain to be elucidated. Thus, SLC38A5 is crucial for melatonin-induced suppression of anoikis resistance and is a promising clinical target in primary bone cancer diagnosis. While our findings provide important insights, several limitations should be considered. First, although our data suggest a clinically relevant role for SLC38A5 in bone cancer progression, its prognostic and therapeutic significance requires further validation in larger, well-annotated patient cohorts. Second, the mechanistic insights presented here are primarily based on a limited number of bone cancer cell models, and future studies employing additional *in vitro* and *in vivo* systems will be necessary to establish the generalizability of these observations. Finally, whether therapeutic targeting of the SLC38A5-glutamine metabolic axis, alone or in combination with melatonin-based strategies, can achieve clinically meaningful efficacy remains an important question for future investigation.

## Conclusion

**Figure 8:**
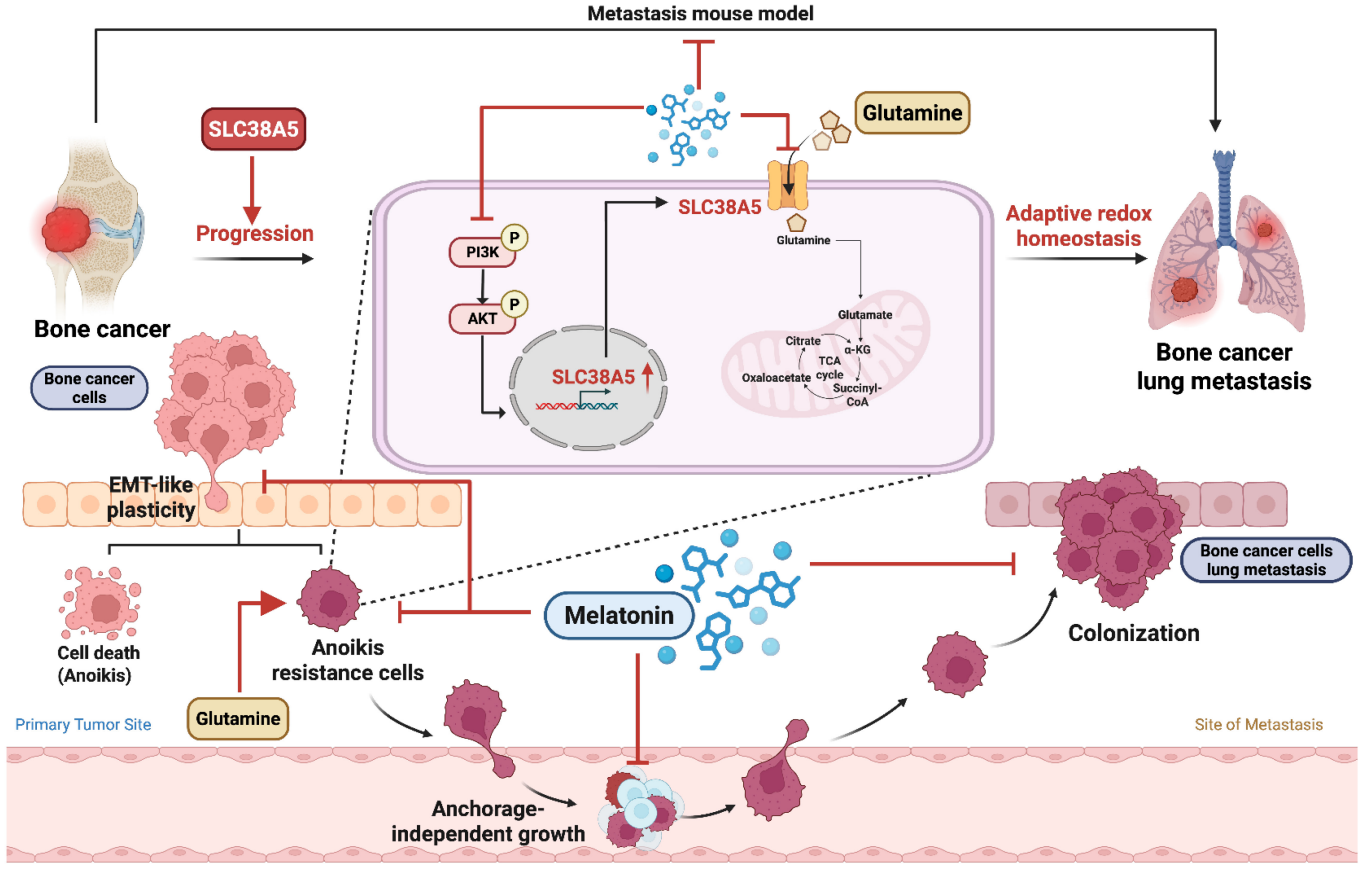
** Schematic model showing that anchorage-independent bone cancer cells depend on SLC38A5-mediated glutamine metabolism to maintain redox balance and survive (Created in BioRender).** Melatonin suppresses anoikis resistance in bone cancer cells by downregulating SLC38A5-dependent glutamine metabolism via inhibition of PI3K-Akt signaling. This metabolic disruption is accompanied by altered redox homeostasis, characterized by increased ROS accumulation and reduced NADPH/NADP⁺ buffering capacity, thereby promoting anoikis and limiting metastasis.

In summary, this study demonstrates that melatonin suppresses anoikis resistance and metastatic potential in bone cancer by inhibiting SLC38A5-dependent glutamine metabolism and PI3K-Akt signaling. Under anchorage-independent conditions, melatonin treatment is associated with disrupted redox homeostasis, as evidenced by increased ROS accumulation and a reduced NADPH/NADP⁺ ratio, effects that are partially reversed by restoring SLC38A5 expression. (Fig. 8). Collectively, these findings reveal a metabolic-redox vulnerability in bone cancer and support melatonin and SLC38A5 as potential therapeutic targets in osteosarcoma and chondrosarcoma.

## Supplementary Material

Supplementary methods and figures.

## Data Availability

The RNA-seq data generated in this study have been deposited in the Gene Expression Omnibus (GEO) database under accession number GSE325221 (https://www.ncbi.nlm.nih.gov/geo/query/acc.cgi?acc=GSE325221). All other data supporting the findings of this study are available from the corresponding author upon reasonable request.

## Abbreviations

CTCs: circulating tumor cells
EMT: epithelial-mesenchymal transition
GDH1: glutamate dehydrogenase
GLS1: Glutaminase 1
OCR: Oxygen consumption rate
SLC38A5: solute carrier family 38 member 5
ROS: reactive oxygen species
