# Supplementary material for: Impaired redox adaptation through SLC38A5-dependent glutamine metabolism contributes to melatonin-mediated anoikis sensitivity and metastasis suppression in bone cancer
Source: Int J Biol Sci. 2026 May 11;22(10):5246–62. doi: 10.7150/ijbs.133256 (PMC13215246; doi:10.7150/ijbs.133256)
Supplement: Supplementary file 1 — Supplementary methods and figures. [file ijbsv22p5246s1.pdf]

Impaired redox adaptation through SLC38A5-dependent glutamine metabolism contributes to melatonin-mediated anoikis sensitivity and metastasis suppression in bone cancer

Bao Tran Nguyen *et al.*

*Corresponding authors:

Chih-Hsin Tang, PhD; *Email:* chtang@mail.cmu.edu.tw

Shun-Fa Yang, PhD; *Email:* ysf@csmu.edu.tw

Supplementary methods

Materials

The phosphorylated (p)-p85 (1:2000; 4228S) and p-Akt (1:2000; 4060S) antibodies were purchased from Cell Signaling Technology, Inc. Antibodies for SLC38A5 (1:500; sc-515703), Akt (1:500; sc-5298) and p85 (1:500; sc-1637) were obtained from Santa Cruz Biotechnology, Inc., the β-Actin (1:5000; GT5512) antibody was purchased from GeneTex International Corporation. GLS antibody (1:2000; HPA036223) and MTT buffer were purchased from Sigma-Aldrich (Merck KGaA). Lipofectamine® 2000 was supplied by Invitrogen (Thermo Fisher Scientific, Inc.). The Novolink Polymer Detection Systems kit was purchased from Leica Biosystems (St. Gallen, Switzerland).

Cell culture

The SW1353, 143B, and MG63 cell lines were obtained from the American Type Culture Collection (ATCC, Manassas, VA, USA), while the JJ012 cell line was kindly provided by Dr. Sean P. Scully (University of Miami School of Medicine, Miami, FL, USA). All cell lines were maintained under culture conditions as outlined in our previous reports [1, 2].

Western blotting

Total cellular proteins were isolated with RIPA lysis buffer and resolved by SDS–PAGE. Following electrophoretic transfer to PVDF membranes, non-specific binding was blocked with 5% skim milk for 1 h. After that, membranes were incubated overnight at 4 °C with primary antibodies and subsequently with secondary antibodies. Chemiluminescent detection (ECL; Merck

Millipore) was performed, and images were acquired using the iBright Imaging System (Thermo Fisher Scientific, Rockford, IL, USA) [3, 4].

Reverse transcription quantitative-polymerase chain reaction (RT-qPCR)

Total RNA was extracted from bone cancer cells following the manufacturer's instructions using the TRIzol® reagent kit (Thermo Fisher Scientific, Inc.). RNA purity was confirmed by A260/A280 ratios of 1.8–2.0, and 1 μg of qualified RNA was reverse-transcribed into cDNA using an oligo-dT primer and reverse transcriptase.

Quantitative PCR was performed with 100 ng of cDNA, sequence-specific primers, and the KAPA SYBR® FAST qPCR Kit (Applied Biosystems; Thermo Fisher Scientific, Inc.), with GAPDH as the normalization control. The following PCR primer sequences were used: SLC38A5, forward: TGGGGCCATGTCCAGTTAC and reverse: GGCAGGATGATTAACACACTGAC; GLS, forward: AGGGTCTGTTACCTAGCTTGC and reverse: ACGTTCGCAATCCTGTAGATTT; and GAPDH forward: ACCACAGTCCATGCCATCAC and reverse: TCCACCACCCTGTTGCTGTA. The following thermocycling conditions were used for PCR: Initial denaturation at 95˚C for 10 min; 40 cycles of 95˚C for 15 sec and 60˚C for 1 min. Amplification conditions were 95 °C for 10 min, followed by 40 cycles of 95 °C for 15 sec and 60 °C for 1 min, performed on a StepOnePlus system (Applied Biosystems; Thermo Fisher Scientific, Inc.) in triplicate.

Supplementary figure

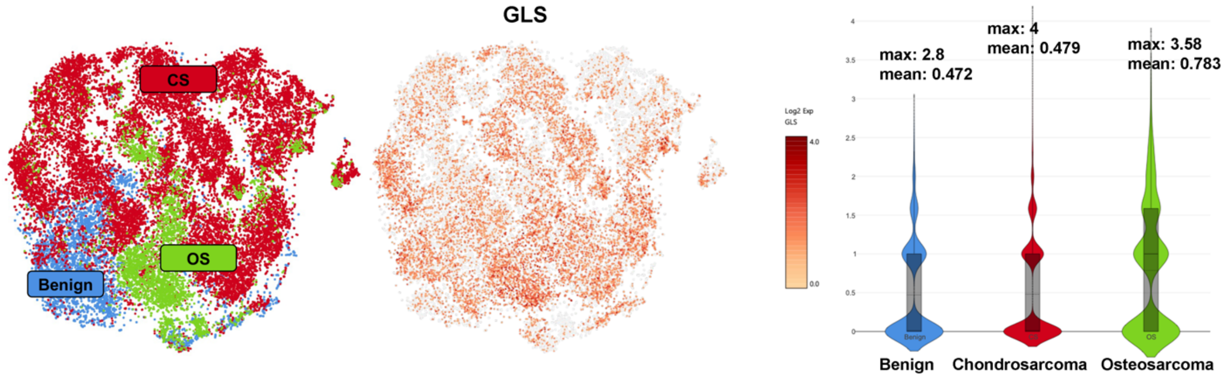

Figure S1. Single-cell RNA sequencing analysis of GLS expression in bone cancer, including chondrosarcoma and osteosarcoma.

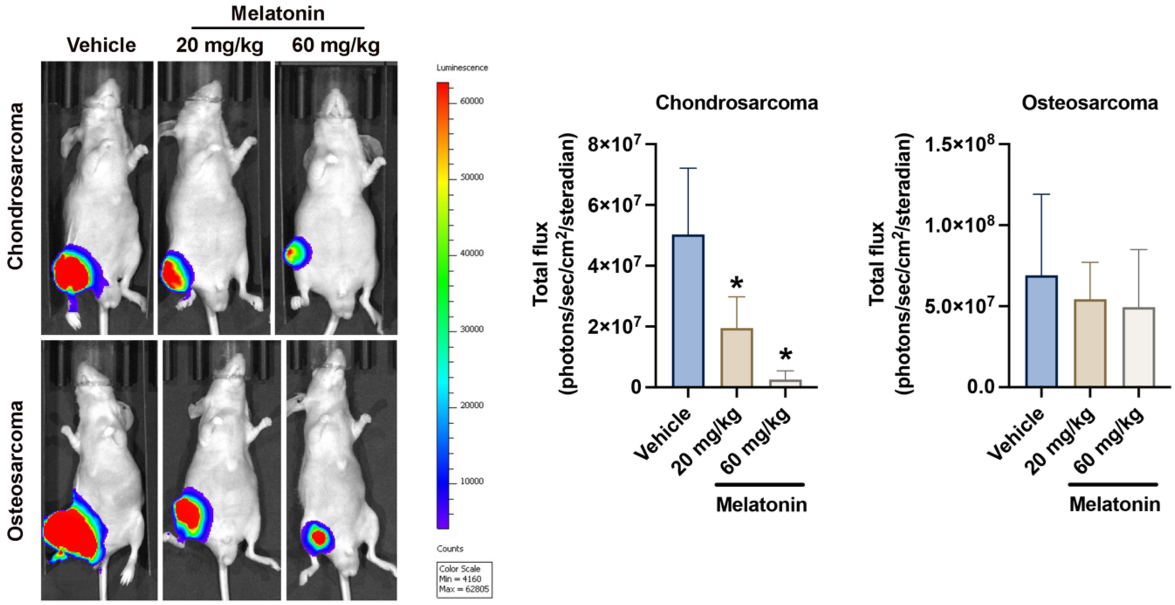

Figure S2. Effect of melatonin on primary tumor growth in bone cancer xenograft model.

References

1. Nguyen BT, Lin CY, Chang TK, Fong YC, Thadevoos LA, Lai CY, et al. Melatonin inhibits chondrosarcoma cell proliferation and metastasis by enhancing miR-520f-3p production and suppressing MMP7 expression. J Pineal Res. 2023; 75: e12872.
2. Trang NTN, Lai CY, Tsai HC, Huang YL, Liu SC, Tsai CH, et al. Apelin promotes osteosarcoma metastasis by upregulating PLOD2 expression via the Hippo signaling pathway and hsa_circ_0000004/miR-1303 axis. Int J Biol Sci. 2023; 19: 412-25.
3. Lee KT, Su CH, Liu SC, Chen BC, Chang JW, Tsai CH, et al. Cordycerebroside A inhibits ICAM-1-dependent M1 monocyte adhesion to osteoarthritis synovial fibroblasts. J Food Biochem. 2022; 46: e14108.
4. Lin CC, Chen KB, Tsai CH, Tsai FJ, Huang CY, Tang CH, et al. Casticin inhibits human prostate cancer DU 145 cell migration and invasion via Ras/Akt/NF-κB signaling pathways. J Food Biochem. 2019; 43: e12902.
